# Clinical features and outcome in paediatric newly diagnosed immune thrombocytopenic purpura in a tertiary care centre

**DOI:** 10.12669/pjms.345.15687

**Published:** 2018

**Authors:** Huma Zafar, Saadia Anwar, Mahwish Faizan, Shazia Riaz

**Affiliations:** 1 Huma Zafar, FCPS. Department of Paediatric Haematology, Oncology and Bone Marrow Transplant, The Children’s Hospital and the Institute of Child Health, Lahore, Pakistan; 2 Saadia Anwar, FCPS. Department of Paediatric Haematology, Oncology and Bone Marrow Transplant, The Children’s Hospital and the Institute of Child Health, Lahore, Pakistan; 3 Mahwish Faizan, FCPS. Department of Paediatric Haematology, Oncology and Bone Marrow Transplant, The Children’s Hospital and the Institute of Child Health, Lahore, Pakistan; 4 Shazia Riaz, MD. Department of Paediatric Haematology, Oncology and Bone Marrow Transplant, The Children’s Hospital and the Institute of Child Health, Lahore, Pakistan

**Keywords:** Immune thrombocytopenia, Immature platelet fraction, Outcome of immune thrombocytopenia

## Abstract

**Objective:**

The study aimed to demonstrate the pattern of clinical presentations and outcome of acute Immune Thrombocytopenia (ITP) in our Centre.

**Methods:**

A descriptive, observational study was conducted by collecting and analysing the data of 103 patients of acute ITP, ageing between 1-14 years, at The Children’s Hospital, Lahore from January 2016 to December 2016. We collected the data regarding age, sex, clinical presentations, history of preceding viral infections, vaccination history, laboratory values, different treatment options used, and response to the treatment concerning complete response, partial response and poor responders. Statistical analysis performed by using IBM SPSS statistics version 20.

**Results:**

We retrospectively, reviewed total 103 patients cases. The median age, at the time of presentation, was 5±3.4 years while mean age was 4.5±2.9 years. The male to female ratio was 1.28:1. Mean platelet count on presentation was 7 x 10^9^/L (range: 0-24). Twenty three (22.3%), patients had the history of preceding illness. Bruises, petechiae, epistaxis and hematemesis remained the common presentations. Six (5.8%) patients showed spontaneous recovery while 97 (94%) patients received treatment for ITP. Overall, 71 (68.9%) showed a response after treatment. Sixty-two patients (59.22%) showed loss of response and received treatment again. Among these patients, thirty-four patients (33%) developed chronic disease.

**Conclusion:**

Majority of patients presenting to our tertiary care centre had severe acute ITP on presentation. After management and follow-up, almost 1/3 of the patients develop chronic disease hence the incidence of developing chronic disease remained high as compared to the other centers.

## INTRODUCTION

Platelets also called as thrombocytes are the critical factors in controlling the bleeding disorders, initiating and maintaining haemostasis. Immune Thrombocytopenia (ITP) is an acquired autoimmune, haematological disorder characterized by isolated thrombocytopenia (peripheral blood platelet count <100x109⁄ l) in the absence of other causative systemic disorders.^1^,^2^ The pathogenesis of ITP remains incompletely understood. The underlying mechanism is thought to be the reduced lifespan of platelets due to antibodies-mediated accelerated destruction by the reticuloendothelial system and abnormal production by bone marrow.^2^,^3^ Previously, it was believed that increased platelet destruction at a rate exceeding production by a compensating bone marrow is solely responsible for ITP, but recent advancements proved that platelet production is also decreased in many patients with ITP.^1^,^4^ This abnormally low count may subsequently manifest itself as an excessive internal or external bleed, easy bruisability (purpura) or oozing of blood from vessels into the skin and mucous membranes (petechiae).^5^

ITP can be classified clinically as newly diagnosed/acute (diagnosis to 3 months), persistent (3 to 12 months) and chronic (lasting for more than 12 months).^6^ It is a self-limiting condition and may not require any treatment. More than 80% of diagnosed children, if left untreated, may undergo spontaneous remission with complete recovery of platelet count within 2-8 weeks.^5^ Patients presenting with no bleed or mild bleed (defined as skin manifestations only, such as bruising and petechiae) are managed with observation alone irrespective of platelet count.^1^ Risk of severe haemorrhage and intracranial bleed is about 3% and less than 1% respectively.^5^,^7^ In all cases with clinically significant bleed, treatment aims to achieve adequate hemostasis and not the normal platelet count. First line drugs therapy includes oral corticosteroids (2mg/kg/day) for two weeks followed by tapering over one week or intravenous methylprednisolone (30mg/kg for 3 days) or a single dose of intravenous immunoglobulin (IVIG) (0.8-1g/kg) or intravenous Rhesus anti-D (50-75 ug/kg).^1^

The objective of our study was to demonstrate the pattern of clinical presentation of paediatric patients of newly diagnosed ITP and to document its outcome in our centre.

## METHODS

A descriptive, observational study was conducted at the Department of Paediatric Haematology/ Oncology & BMT, The Children’s Hospital & The Institute of Child Health from January 2016 to December 2016. We studied over 103 patients meeting the inclusion criteria, i.e., age between 1-14 years, presented with the diagnosis of acute ITP, did not take any previous treatment and remained on regular follow up over a period of one year. Paediatric patients less than one-year-old or those, having chronic ITP or showing incomplete data were excluded from the study. The institutional review board approved the study. We collected the data keeping in accordance of age, sex, clinical presentations, history of preceding viral infections, vaccination history within 8-12 weeks, seasonal variations, laboratory values, different treatment options used, and response to the treatment in terms of complete response, response and poor responders. Response after initial treatment was measured by platelet count at 48 hours of therapy in case of IVIG, I/V steroids and I/V anti-D, and at two weeks in case of oral steroids. Statistical analysis performed by IBM SPSS statistics version 20. Patients were followed for one year from the time of presentation to find out the number of patients developing chronic disease. Platelet counts were monitored at three months, six months and 12 months from the time of diagnosis.

### Operational definitions

***ITP:*** Isolated thrombocytopenia (peripheral blood platelet count < 100 x109⁄ l) in the absence of other causative systemic disorders.^1^,^2^

***Acute ITP:*** Newly diagnosed case up till 3 months.^6^

***Persistent ITP:*** Three months from diagnosis till 12 months.^6^

***Chronic ITP:*** ITP lasting for more than 12 months.^6^

***Complete response:*** A platelet count ≥100 x10^9^/L, measured on 2 occasions 7 days apart and the absence of bleeding.^1^

***Partial response:*** A platelet count ≥30 x 10^9^/L or more than two-fold increase in platelet count from baseline, measured on two occasions, seven days apart and the absence of bleeding.^1^

***No response:*** A platelet count < 30 x 10^9^/L or a less than 2-fold increase in platelet count from baseline or the presence of bleeding. Platelet count must be measured on two occasions more than a day apart.^1^

***Loss of response:*** A platelet count <30 x 10^9^/L or a less than 2-fold increase in platelet count from baseline or the presence of bleeding, after achieving an initial complete/partial response. Platelet count must be measured on two occasions more than a day apart.^1^

## RESULTS

The record of 103 patients was reviewed retrospectively during the study period, diagnosed as acute Idiopathic Thrombocytopenic Purpura. The median age, at the time of presentation, was 5±3.4 years while mean age was 4.5±2.9 years. Amongst 103 patients, 58 (56.31%) patients were male while 45 (43.6%) patients were females. The male to female ratio was 1.28:1 (Table-I). Mean platelet count on presentation was 7x10^9^/L (range: 0-24). At the time of presentation, the platelet count did not vary between the two sexes (p>0.05). Regarding age, the children above 10 years presented with least platelet count (Table-II).

**Table-I:** Distribution of age and mean platelet count at presentation (n= 103).

Age group	Male	Female	Mean Platelet Count (10^9^/L)
12 month – 5years	30 (29.1%)	22 (21.3%)	09
>5 years – 10 years	21 (20.3%)	17 (16.5%)	13
>10 years – 14 years	07 (6.7%)	06 (5.8%)	07

Total	58 (56.3%)	45 (43.6%)	

**Table-II:** Platelet counts on presentation (n=103).

Platelet counts (10^9^/L)	n (%)
<10,000	43 (41.7)
10,000 – 20,000	42 (40.8)
>20,000	18 (17.5)

Furthermore, 23 (22.3%) patients had the history of preceding illness; 13 (12.6%) with upper respiratory infections, 9 (8.7%) with acute gastroenteritis and 1 (1.0%) with varicella zoster infection. None of the patients had received vaccinations, prior to developing symptoms of ITP within 8-12 weeks. Bruises, petechiae, epistaxis and hematemesis remained the common presentations. On clinical examination, 92 (89.3%) patients were reported to have bruises, 81 (78.6%) patients with petechiae, 33 (32.0%) with epistaxis, 13 (12.6%) with hematemesis, and five (4.9%) patients with menorrhagia (Table-III). Sixty-two (60.2%) patients were diagnosed by clinical presentations, thirty-two (31.0%) patients after bone marrow examination and nine (8.7%) patients by immature platelet fraction (IPF).

**Table-III:** Clinical features of patients at presentation.

Clinical features	No. of patients n (%)
Bruises	92 (89.3)
Petechiae	81 (78.6)
Epistaxis	33 (32.0)
Hematuria	13 (12.6)
Melena	3 (2.9)
Menorrhagia	5 (4.9)
ICB	1 (1.0)

Six (5.8%) patients showed spontaneous recovery while rest of the patients were treated with one or more of the option mentioned above, at one time or more. Overall, 97 (94%) patients received treatment for ITP. Treatment was given only to symptomatic patients with significant bleeding diathesis. Treatment type was based on severity of symptoms. Patients with severe life threatening active bleed were given combination treatment with IV Solomedrol, IVIG and platelet transfusion. Oral steroids were 1^st^ line of treatment in stable patients, while IVIG and Anti D (for rhesus positive patients) was 1^st^ line treatment for patients with active bleed admitted through emergency. A total of 32 (31.1%) patients received IVIg, 30 (29.1%) received oral steroids, while 22 (21.3%) received I/V steroids, 5 (4.9%) received both I/V steroids and IVIg, and 8 (7.8%) received anti D (Table-IV).

**Table-IV:** Treatment given to ITP patients and Response (n=103).

Treatment	Patient received treatment n (%)	Partial response shown
IVIG	32 (31.1%)	24 (75.0%)
Methylprednisolone	22 (21.4%)	16 (72.7%)
Oral steroid	30 (29.1%)	23 (76.7%)
Anti-D	8 (7.8%)	3 (37.5%)
Combination therapy	5 (4.9)	5 (100%)
No treatment	6 (5.8%)	-

Overall, 71 (68.9%) showed a response after treatment. All patients were followed clinically and with their platelet counts for total of 12 months. Follow up was initially weekly after an acute episode to document response/no response and latter on monthly basis for loss of response. Sixty-two patients (59.2%) showed relapse of thrombocytopenia and received treatment again, once or more. Among these patients, 34 (33%) patients developed chronic disease. Most of the patients (n=9, 70%) from the age group 10-14 years did not achieve remission at the end of one year. Interestingly, age above 10years was observed to play a significant role in developing chronicity of the disease (p<0.05). Besides, female patients above 10 years showed higher risk of developing chronic ITP than male patients (p<0.05). However, after exclusion of patients above 10 years, the incidence of developing chronic disease was found to be similar between two genders (p>0.05).

## DISCUSSION

Regarding the epidemiological data of acute paediatric ITP in our centre, the results varied with those of others. In our study, the boys outnumbered the girls, although two studies conducted previously in Pakistan, by Farid et al. and Mushtaq et al. observed female predominance in patients with acute ITP.^8^,^9^ Vast majority of our patients presented had a clinically significant bleed that warranted medical treatment while a large study group reported that more than half of the patients had no or only mild bleed.^10^ A reason might be that acute ITP is also treated by general pediatricians and only complicated cases are referred to Pediatric hematologist/oncologists. Our data was collected at a tertiary care center and thus shows complicated initial presentation for this cohort.

In our study, preceding viral illness found in only 22% patients while a study in China demonstrated the history of viral illness in 74% patients.^11^ This marked difference can probably be due to poor historians or the fact that people in low-income countries like Pakistan do not register minor viral illnesses and frequently overlook them.

We observed that more than half of our patients (60.2%) were diagnosed by clinical features only (bone marrow aspiration, biopsy or immature platelet fraction not done) and follow up of those patients over a period of one year showed no change in the diagnosis. Bone marrow biopsy does not seem to be mandatory for the diagnosis of acute ITP and this matches with the general British and American guidelines and conclusions of other studies.^12^,^13^

In our study, children above 10 years presented with severe thrombocytopenia. Five such patients received combination therapy (I/V Methylprednisolone and IVIG). A combination therapy given to 11.6% of the patients in a study (I/V Methylprednisolone and IVIG) recommended it for older children of acute ITP. While in our study, only 5 (4.9%) patients received both the drugs simultaneously. All these patients, who received combination therapy showed a response after initial treatment but 12 months follow up revealed that only 40% achieved complete remission. We observed two factors; age above 10 years and female gender which play significant role in developing chronic ITP, which is similar to the results of a study conducted by Evim et al.^14^ In our study, amongst 103 patients, 34 (33%) patients developed chronic disease while in a study, by Champatiray et al., 36 (14%) patients showed chronic disease. Similarly in another study by Mushtaq et al., out of 95 children, only 5 (5.3%) patients developed chronic ITP.^9^,^15^ The high rate of chronicity might be due to complicated initial presentation, as suggested by Heitink-Pollé KM et al, female gender, older age at presentation, absence of preceding infection or vaccination were identified as risk factors for developing chronic ITP.^16^ However this warrants further investigation.

Some studies published in literature show significant association of Acute ITP with MMR vaccination.^17^ In our study, none of the patients had a history of receiving vaccines in last 8 to 12 weeks. Studies conducted in five health care systems, on 197 cases of ITP also reported that there is no significant increase in the risk of developing ITP after early childhood vaccines. While a systematic review of 12 studies showed that post MMR vaccines ITP is a rare phenomenon, reporting only 2.6 cases per 100,000 vaccines doses.^18^ Besides, all these cases presented in a milder form showing a self-limiting pattern. Having discussed these results and keeping in view the high risk of developing infections, which are preventable with these vaccines, association of ITP with the childhood vaccines should not be used as a reason for limiting the use of vaccines in a developing country like Pakistan, thus supported by the conclusion of the study by Cecinati V et al.^19^

Our study shows varied presentation, male predominance without significant preceding history of viral infection or vaccination. However this is a single center study carried out in a tertiary care referral center. Collaborative groups and national guidelines do not exist to manage ITP in children, which are much needed. Multicenter, prospective randomized control trials are needed, using standardized management protocols, involving large cohort of patients to evaluate exact disease behavior in our country.

## CONCLUSION

Majority of patients presenting to our tertiary care centre had severe acute ITP on presentation. After management and follow-up, almost 1/3 of the patients develop chronic disease hence the incidence of developing chronic disease remained high as compared to the other centers. Pediatric ITP diagnosis is mainly clinical and does not require bone marrow biopsy for confirmation Further prospective studies are required to identify different risk factors and to compare the results of different treatment options in use.

### Authors’ Contribution

**HZ:** Conceived & designed the study, data collection and manuscript writing

**SA:** Assisted in writing manuscript, reviewed manuscript.

**MF:** Designed the study and statistical analysis.

**SR:** Reviewed the literature, reviewed manuscript, proofreading.
